# Monitoring the Land Subsidence Area in a Coastal Urban Area with InSAR and GNSS

**DOI:** 10.3390/s19143181

**Published:** 2019-07-19

**Authors:** Bo Hu, Junyu Chen, Xingfu Zhang

**Affiliations:** Surveying Engineering, Guangdong University of Technology, Guangzhou 510006, China

**Keywords:** time-series InSAR, subsidence, GNSS, coastal urban area

## Abstract

In recent years, the enormous losses caused by urban surface deformation have received more and more attention. Traditional geodetic techniques are point-based measurements, which have limitations in using traditional geodetic techniques to detect and monitor in areas where geological disasters occur. Therefore, we chose Interferometric Synthetic Aperture Radar (InSAR) technology to study the surface deformation in urban areas. In this research, we discovered the land subsidence phenomenon using InSAR and Global Navigation Satellite System (GNSS) technology. Two different kinds of time-series InSAR (TS-InSAR) methods: Small BAseline Subset (SBAS) and the Permanent Scatterer InSAR (PSI) process were executed on a dataset with 31 Sentinel-1A Synthetic Aperture Radar (SAR) images. We generated the surface deformation field of Shenzhen, China and Hong Kong Special Administrative Region (HKSAR). The time series of the 3d variation of the reference station network located in the HKSAR was generated at the same time. We compare the characteristics and advantages of PSI, SBAS, and GNSS in the study area. We mainly focus on the variety along the coastline area. From the results generated by SBAS and PSI techniques, we discovered the occurrence of significant subsidence phenomenon in the land reclamation area, especially in the metro construction area and the buildings with a shallow foundation located in the land reclamation area.

## 1. Introduction

Traditional geodetic methods represented by levelling or total station geometry are point-based measurements. In the task of deformation monitoring, the usual operation process is: Firstly, select representative sites in the monitoring area and set up monitoring stations (observation station or observation markers), and then set the monitoring period for a single or continuous observation. From the original geodetic workflow, we can find that the traditional geodetic survey method has two significant characteristics: The first is the necessity of setting up the observation stations and observation markers. The other one is the long working time-consuming of each period of observations. The former characteristic leads to an insufficient spatial sampling rate of monitoring results which is unable to discover new deformation areas from the monitoring results effectively, especially when the monitoring scope is relatively extensive, and the latter one limits the temporal sampling rate of monitoring results of the station or the observation marker. Therefore, in the task of early identification and monitoring of a deformation area, we need a method that can conduct extensive observation to the monitoring region in a short period, and provide a reliable result for early identification and continuous observation of the region where geological disasters occurred. Interferometric Synthetic Aperture Radar (InSAR) has the advantages of an extensive monitoring area, high temporal resolution, and all-weather monitoring capability [1,2,3,4,5,6]. The time-series InSAR (TS-InSAR) technology effectively weakens the key factors that affect the reliability of InSAR monitoring results: Atmospheric effects and time-space decoherence [1,7]. InSAR has been widely used by researchers in different fields of research over the past decade. InSAR shows significant advantages in various fields of geoscience, including seismicity research [8,9,10,11], volcanic research [12], artificial building deformation monitoring [13], and surface deformation caused by underground resource exploitation [14,15]. In the case of urban areas research, Yang Zhang et al. used RADARSAT-2 satellite data to monitor the land subsidence of Wuhan, China, with Small BAseline Subset (SBAS) [16]. Xiaoqiong Qin et al. used the TerraSAR-X image to monitor land subsidence along the subway in the urban area of Shanghai, China [17]. Matthew North et al. studied the response of seasonal soil movement along the British railway and road with the PSI method [18]. Bing Xu et al. studied the land reclamation area with ENVISAT data [19]. In these examples, InSAR technology provides researchers with a reliable way of generating land deformation velocity fields.

The SBAS method is a kind of InSAR time series surface deformation inversion technology proposed by Berarndino [1] in 2002. To the InSAR differential interferogram, the stability of the interferometric phase is affected by the temporal baseline of the interferometric pair and the spatial baseline. Different from the traditional Differential InSAR (D-InSAR) method, SBAS is characterized by reducing spatial decoherence and screening out the interferometric pairs with higher coherence by limiting the time baseline and the spatial baseline. One applies the small baseline differential interferogram to the surface deformation inversion model, which can obtain the deformation time series of the coherent target, while ensuring that the factors affecting the interferometric quality of the interferogram are estimated and removed. The PSI method mainly focuses on the target that maintaining a high coherence in the interferometric data set, which is called “permanent scatterers (PS)” [9,20,21]. PS usually targets on the artificial buildings and bare stones. PS provides stable interferometric phase coherence. The PSI method directly executes the inversion model on the unwrapped differential interferometric phase, so that it can effectively avoid the unwrapping error caused by the traditional InSAR technology in the phase unwrapping step.

We use SBAS and PSI to study ground deformation in Shenzhen and HKSAR. In Shenzhen, we mainly study the Qianhai area with the risk of subsidence [19] using both the SBAS and PSI methods. In HKSAR, we use the SBAS method and Global Navigation Satellite System (GNSS) static network which consists of a series station that belongs to Hong Kong Continuously Operating Reference Stations, CORS (SatRef), run by Lands Department of HKSAR. The observation data observed by the GNSS station are transformed into Receiver INdependent EXchange (RINEX) format. Hi-Target Geomatics Office (HGO) software was used to generate the geodetic coordinates and the geodetic height under the WGS-84 coordinate system using the GNSS static network observation mode. The baseline solution solving step uses the SP3 post-accuracy ephemeris data released from International GNSS Service (IGS) to provide satellite coordinate information to maximize the accuracy of the baseline solution. 

## 2. Overview of Study Area

**Figure 1:**
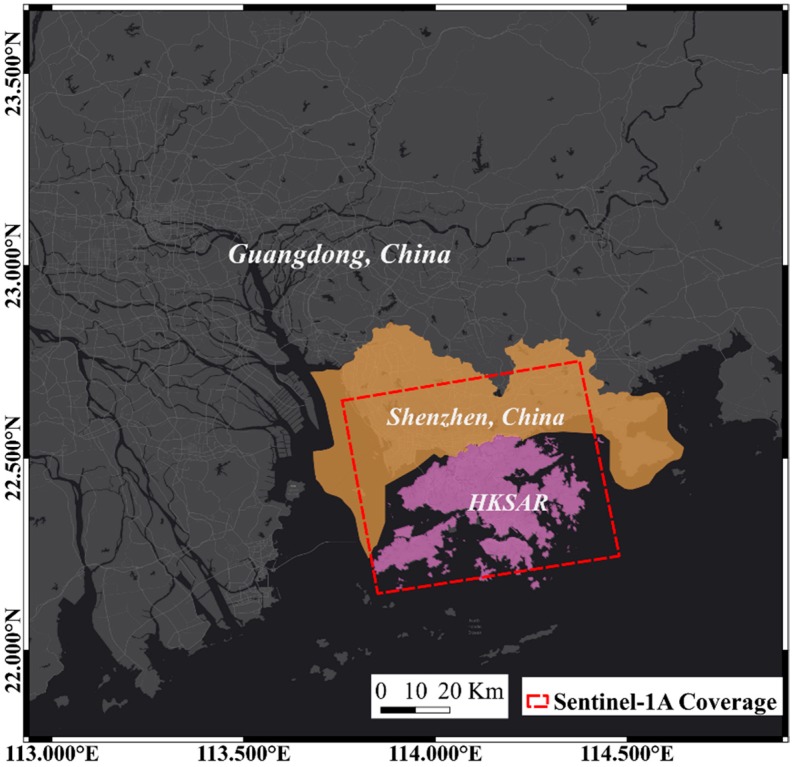
Location of the study area and the coverage of cropped Sentinel-1A SAR image.

The study area includes Shenzhen, China and the HKSAR bordering the south (Figure 1). Shenzhen is located in the southern part of Guangdong Province, China. As one of the special economic zones of China, Shenzhen has carried out much urban construction since 1980. In recent years, the construction area of the city has been transferred from Luohu district in the central part of Shenzhen city to Nanshan district in Qianhai district in the west. While HKSAR started urbanization earlier, it also carried out a large number of land reclamation projects to acquire land used for construction. A famous example is the Hong Kong International Airport, HKIA (Rose Garden Project). The construction project of the HKIA started in 1992 and was commissioned in 1998. The third runway and associated facilities will be constructed on the north side of the existing airport. The land reclamation project for the runway expansion began in 2016 [22]. In the rapid urbanization construction of these two cities, the land reclamation area is widely distributed in the study area. Since the special economic zone was built in Shenzhen in the 1980s, the land reclamation area reached 69 km^2^, while the land reclamation area in HKSAR is 70 km^2^. Qianhai district is the hot-spot area for infrastructure construction in Shenzhen [23,24,25]. Since 2006, the Qianhai district has begun reclamation. The land reclamation area of Qianhai district reached 9.9 km^2^ in 2009. 

Shenzhen runs eight subway line operators, with a total mileage of 285 km. It is estimated that by 2022, the Shenzhen Metro will reach 16 operating lines with a mileage of 596 km. The HKSAR metro system has a mileage of 264 km with 11 operating lines and average daily passenger flow of 5.59 million (2016) [26]. 

## 3. Data and Methods

Both GNSS technology and InSAR technology are space geodetic techniques which are capable of all-weather observation. InSAR technology uses space-based SAR sensor to obtain SAR images, then generates the displacement phase through performing a differential operation by acquiring the interferometric phase of the coherence target in the SAR dataset [12,14]. Therefore, InSAR technology has the advantage of a high spatial resolution, which is suitable for locating the area that assumes land deformation. However, the satellite has a revisit cycle which is usually about ten days, so InSAR technology cannot achieve high time resolution monitoring. In principle, the PSI method and the SBAS method are used for different kinds of targets in the inversion model, the spatial distribution characteristics between SBAS and PSI inversion results would be different [7]. In areas with extensive urbanisation, more PS points will be selected in this type of study area. 

The GNSS technology can reach a very high temporal resolution observation by setting the sampling interval up to the second level due to the observed relationship using the ground-mounted GNSS antenna [27]. CORS stations which are deployed in urban areas can be observed in unattended conditions by deploying permanent GNSS antennas and receivers. The GNSS technology can form a certain degree of complementarity with the InSAR technology, which has a satellite revisit cycle. 

**Figure 2:**
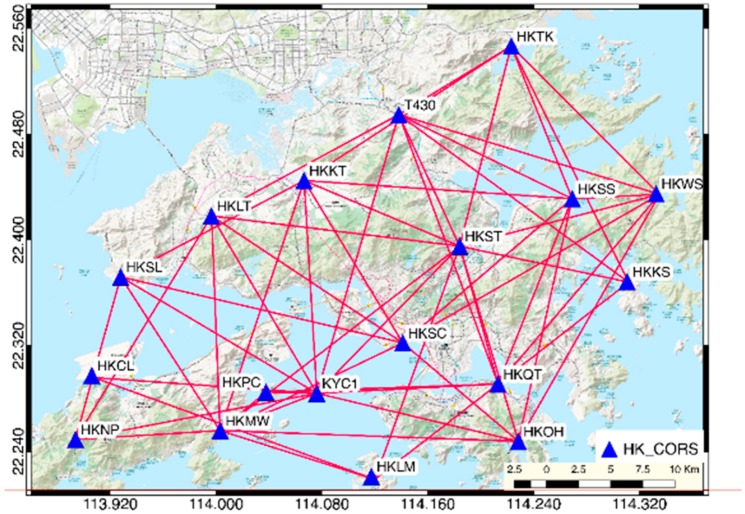
HK Continuous Operating Reference Station (CORS) network (SatRef).

The GNSS observation data we used in this paper was published by the satellite positioning reference station network in Hong Kong name “SatRef” (Figure 2) operated by the Survey and Mapping Office (SMO), Hong Kong Land Department. The reference station network consists of 18 continuous operating reference station (CORS) distributed in the HKSAR, including 16 reference station and two integrity monitoring station. The data services of The Hong Kong Satellite Positioning Reference Station was launched on February 4, 2010.

The SAR image dataset used in this research is a total of 34 frames of SLC (Single Look Complex) SAR images (Table 1) which generate in the Interferometric Wide Swath (IW) mode from the Sentinel-1A satellite operated by European Space Agency (ESA). To minimize the orbital error in the interferogram, the SLC image data orbital parameters are provided with the post-accurate orbit data (POE product) provided by the ESA. Moreover, the SAR image data were resampled from the original 5 m × 20 m (range resolution × azimuth resolution) to a ground resolution of 20 m × 20 m (range resolution × azimuth resolution) using multi-looks processing. 

**Figure 3:**
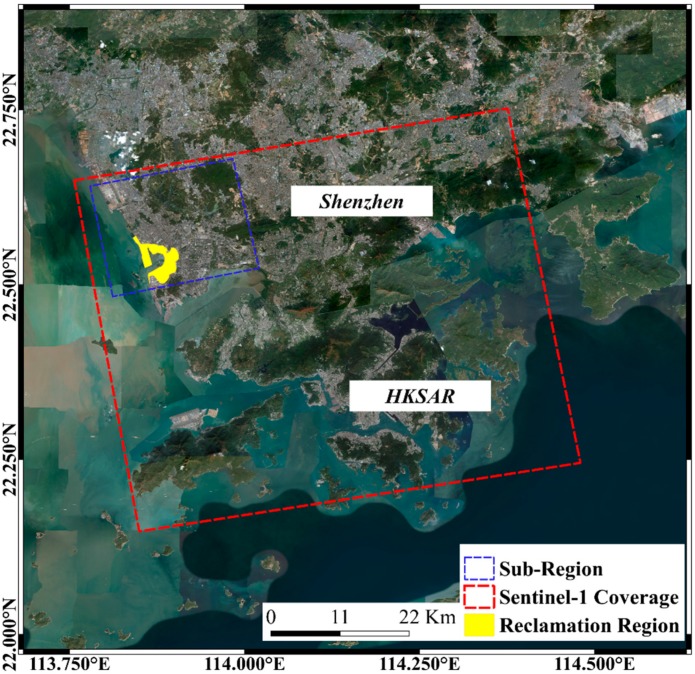
SAR data coverage of the study area.

We intercept the sub-region SAR image data set for PSI data processing (Figure 3). The velocity distribution map, displacement time series distribution of the SBAS inversion results in the sub-region part are export as a vector data for spatial analysis.

In this research, the AW3D digital elevation model was used to remove the topographic phase, which is one of the critical steps in the interferogram generation workflow. The AW3D digital elevation product is produced using panchromatic images acquired from the Panchromatic Remote-sensing Instrument for Stereo Mapping (PRISM) sensor of ALOS satellites. If one uses AW3D data to provide elevation data for inversion to generate the topographical phase of the study area; then a differential operation would be executed on the inverted topographic phase and the interferometric phase to achieve the purpose of removing the topographic phase in the interferometric phase.

**Figure 4:**
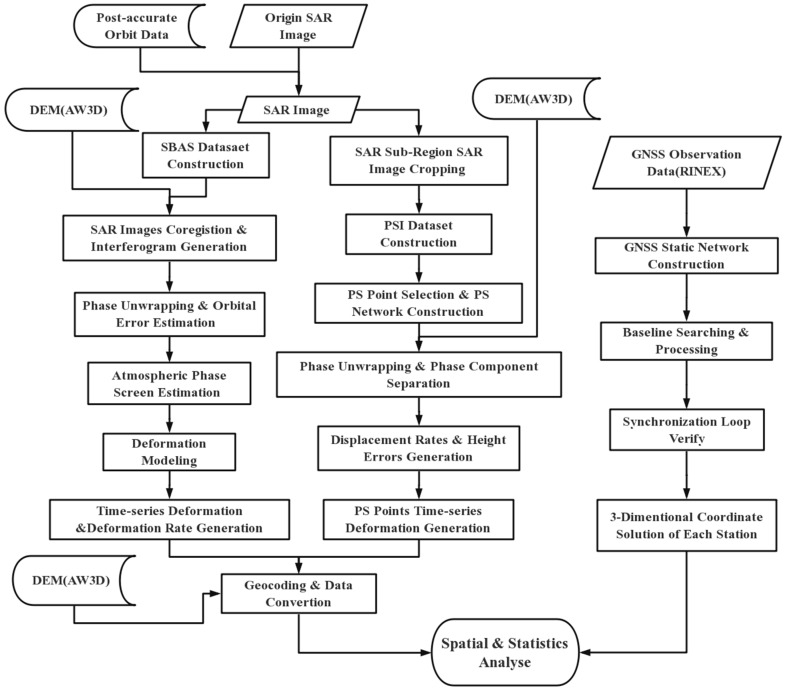
Data processing workflow with SBAS, PSI, and GNSS.

Because there is a large amount of vegetation coverage in the study area, the SAR sensor mounted on the Sentinel-1 satellite works in the C-band with medium ground penetrability. Using the branch cutting or least square phase unwrapping method, it is possible to generate significant unwrapping errors, hence we use the minimum cost flow method for phase unwrapping in the SBAS model (Figure 4). In this research, we use the Delauny Minimum Cost Flow (Delauny-MCF) method to phase unwrap the coherent targets in each interferogram [28,29,30,31,32]. This method constructs a Delaunay triangulation as the unwrapped network. Defining the costs on arcs between two adjacent coherent targets, according to the phase gradient of this pair of adjacent coherent targets, the optimal solution of the cost function of this network can be solved. Moreover, the optimal phase unwrapped path can be generated at the same time [28].

(1) $$\min\left\{\underset{i,j}{\sum }\varpi_{i,j}^{\left(x\right)}{\left|\Delta \varphi_{i,j}^{x}-\Delta \phi_{i,j}^{x}\right|}^{p}+\varpi_{i,j}^{\left(y\right)}{\left|\Delta \varphi_{i,j}^{y}-\Delta \phi_{i,j}^{y}\right|}^{p}\right\}$$

In (1), Δ denotes a gradient along the azimuth and line of sight (LOS) respectively, $\varpi_{i,j}^{\left(y\right)}$ and $\underset{i,j}{\sum }\varpi_{i,j}^{\left(x\right)}$ defined as the weight and the sum of all suitable rows *i* and column *j*. The Delaunay-MCF method is used to enable the unwrapping path to avoid the more low-coherence regions that may contain phase jumps, which could lead to unwrapping error. 

(2) $$\delta \varphi_{m}^{\left(LP\right)}\left(x,r\right)\approx \frac{4\pi }{\lambda }\left[d^{\left(LP\right)}\left(t_{IE_{m}},x,r\right)-d^{\left(LP\right)}\left(t_{IS_{m}},x,r\right)\right]+\frac{4\pi }{\lambda }\frac{b_{m}\Delta z^{\left(LP\right)}\left(x,r\right)}{r\sin\vartheta }\;+\left[\varphi_{atm}\left(t_{IE_{m}},x,r\right)-\varphi_{atm}\left(t_{IS_{m}},x,r\right)\right]+\Delta n_{m}^{\left(LP\right)}\left(x,r\right)$$

We can express the low-frequency part of a coherent pixel in interferogram no.m as Equation (2). λ stands for wavelength, $b_{m}$ stands for the space baseline of the interferometric pair and ϑ stands for the incidence angle of the radar signal of the coherent pixel. $d^{\left(LP\right)}$ represents the deformation component of the interferometric phase $\delta \varphi_{m}^{\left(LP\right)}$ of the coherent pixel which image coordinate is (x,r).$\varphi_{atm}\left(t_{IE_{m}},x,r\right)-\varphi_{atm}\left(t_{IS_{m}},x,r\right)$ represents the atmospheric component between the master image and the slave image and $\Delta n_{m}^{\left(LP\right)}$ stand for the noise term of the interferometric phase. In the process of radar signal propagation in the air, due to the influence of ionosphere and resonance caused by water vapor in the air, a signal propagation delay or path bending in the range is caused. The regional phase changes in each SAR image due to this reason are called atmospheric phase screens (APS). To the interferogram, both the influence of the atmospheric phase and the error in satellite orbit error are low-frequency signals with a typical size of 1km [1]. Therefore, a high-pass filter with the ground range of 1km will be executed in the SBAS deformation inversion model to weaken the errors from the atmosphere and orbit. In this research, we use a linear model to invert the deformation.

## 4. Data Processing and Result

In this research, RINEX observation data was collected from 18 stations with 26 periods in the CORS network (Figure 2). The sampling interval is 30 s, and the observation time is 24 h. In this research, we used the observation mode of GNSS static network for data processing. The average length of the searched baseline is set to 20 km; all the baseline solutions are none ionosphere combination fixed solutions. The tolerance of the 3-dimension components of the synchronization loop is:

(3) $$W_{x}=W_{y}=W_{z}\leq \frac{\sqrt{3}}{5}\delta$$

**Figure 5:**
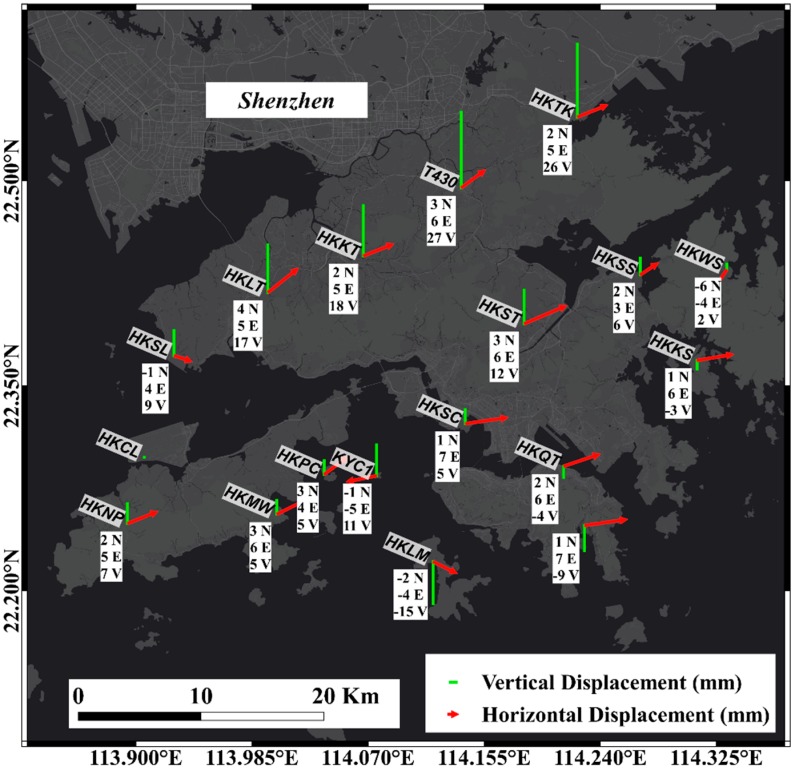
Horizontal and vertical displacement of the Hong Kong CORS network. The arrows and the vertical lines represent the direction and the magnitude of displacement of the station.

Finally, we calculate the general change trend of the reference station during the monitoring period through the static network (Figure 5).

**Figure 6:**
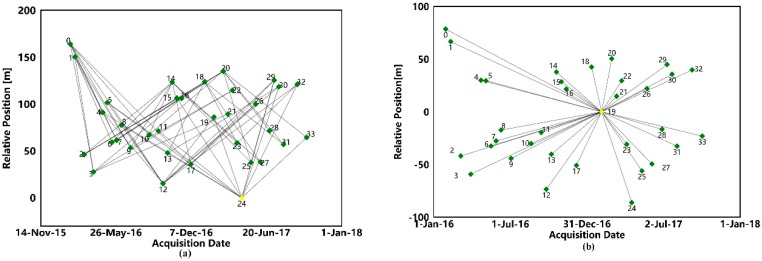
(**a**) Is SBAS interferometric dataset and (**b**) is a PSI interferometric dataset.

**Figure 7:**
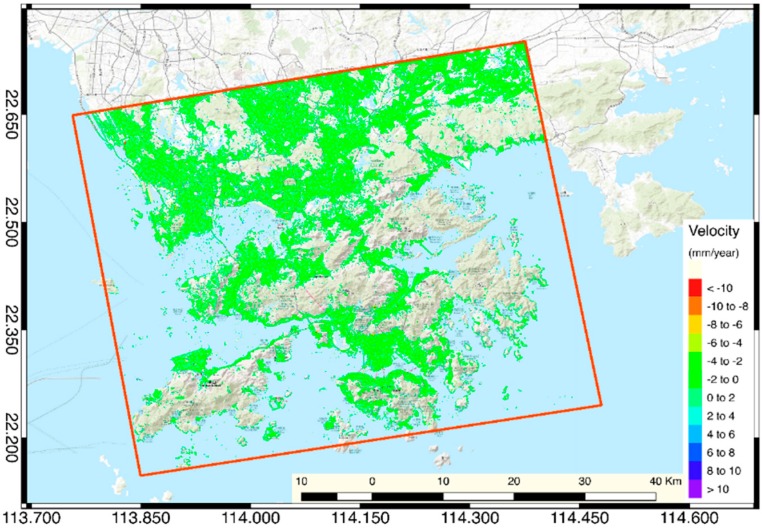
Average vertical velocity distribution of SBAS inversion.

The Sentinel-1A satellite SAR image we used in this research had a revisit period of 15 days. Therefore, the temporal baseline of the interferometric data set used for the inversion of the SBAS method was set to 40–360 days. The threshold of the spatial baseline is 55% of the critical baseline, which is 5889.640 m. In the data processing step, we used the Delauny-MCF method with the phase unwrapping threshold set to 0.47. After removing the interferogram which has significant unwrapping error and an orbit error from the initial interferometric data set, 62 pairs of interferometric pairs were reserved for SBAS data process (Figure 6a). The average elevation accuracy of the SBAS inversion results (Figure 7) is 3.6 mm.

**Figure 8:**
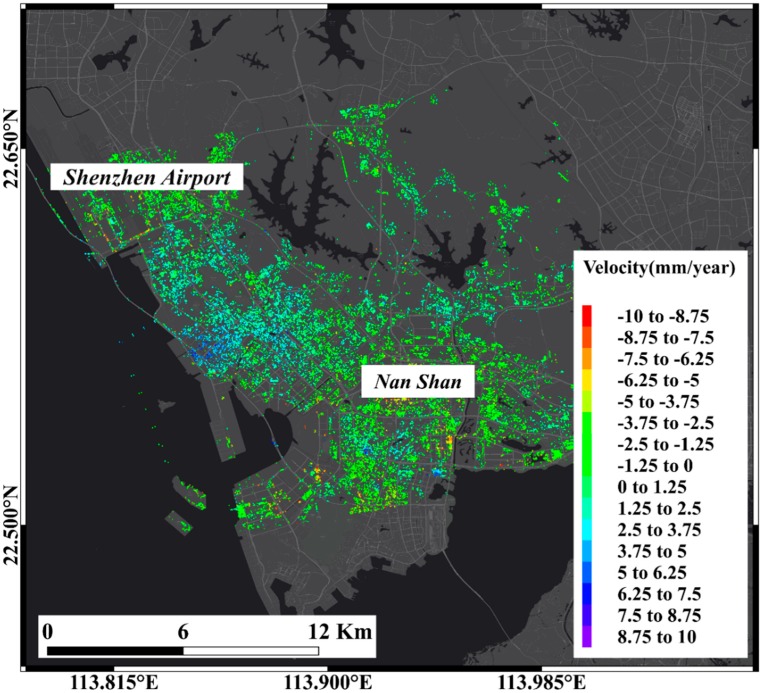
Permanent Scatterer (PS) point in Qianhai district, Shenzhen.

In this research, we use Sentinel-1A SAR data with the PSI method to investigate the coastal area of Qianhai District of Shenzhen and the eastern part of Shenzhen as the research area at the same time. In the PSI method, as long as the baseline length of the interferometric pair is not greater than the critical baseline the baseline of the interferometric pair is not limited, the method of composing the interferometric data set (Figure 6b) using the same main image is adopted in this paper. Alternative PS points are filtered using a coherence threshold method, setting 0.75 as the coherence threshold to extract the PS point in the interferogram of the interferometric data set, a total of 44,464 PS points (Figure 8) were screened out in the study area with an average inversion elevation accuracy of 6.6 mm. 

**Figure 9:**
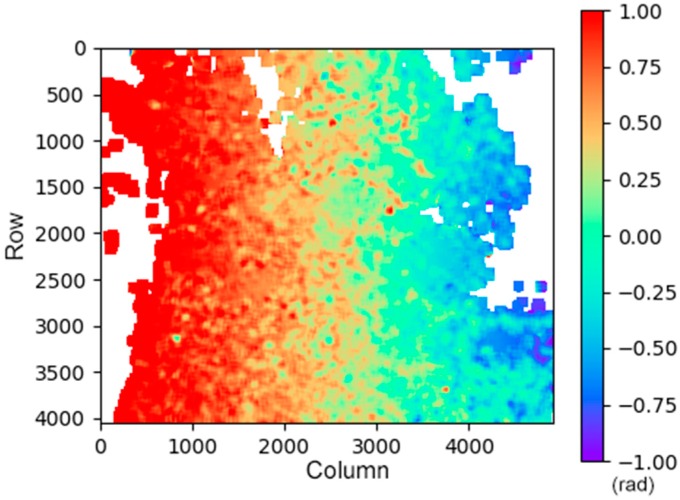
Atmospheric Phase Screens (APS) and orbit error component (low-frequency component) of the master image (2017/4/17) of SBAS dataset.

In the inversion model of SBAS, the inversion of the deformation variables in the model was performed while inverting the atmospheric influences in the inversion model (Figure 9). 

**Figure 10:**
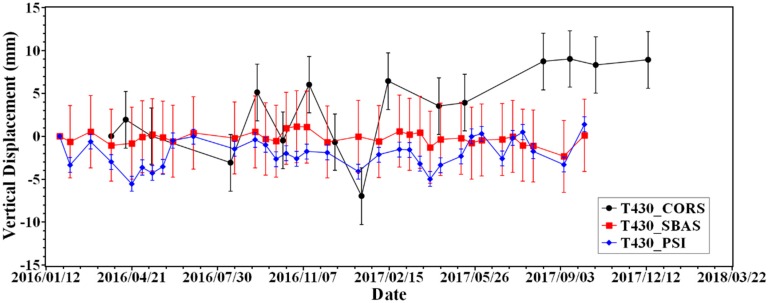
Time series generated by three different solutions on T430 station.

The three-dimensional free network adjustment was performed with the HKCL station as a fixed point after all the simultaneous observation closure differences met the tolerance requirements, and the three-dimensional coordinates of the remaining stations were obtained. We projected the InSAR results from Line of Sight (LOS) to the normal direction of WGS-84 ellipsoid on T430 station. From the time series of variations on this station (Figure 10), we found that because of the lack of accuracy, the GNSS result shows more jumps, although it has advantages of a higher temporal resolution. However, the InSAR result which was generated by two different methods shows consistency both in magnitude and the trend of variations. 

## 5. InSAR in Coastal Area

**Figure 11:**
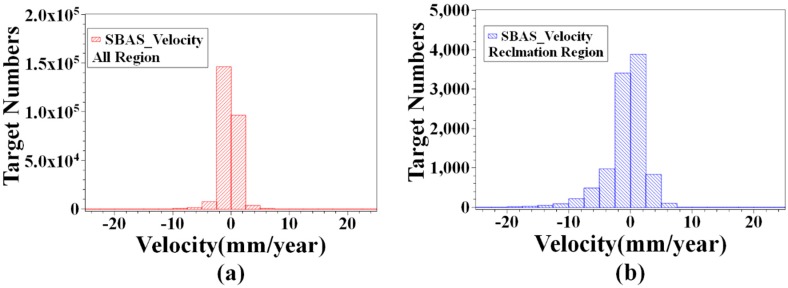

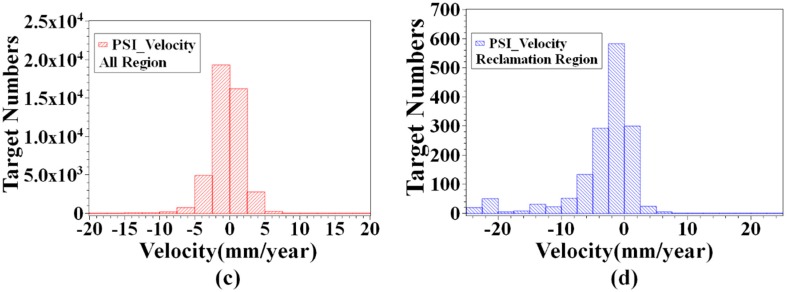
SBAS and PSI inversion result histogram in the sub-region and the land reclamation region.

We used the historical archival image data from optical satellite imagery to determined the reclamation area between the coastline of 1999 which before the land reclamation project and the coastline of 2016 which the land reclamation project was completed in the sub-study area. The sub-region covers 455 km^2^; the land reclamation region covers 22.3 km^2^, which covers about 5% of the sub-region area [19]. There are 255,433 targets in the SBAS inversion results, of which 702 targets have a displacement velocity greater than 10 mm/year, accounting for 0.3% (Figure 11a,b). In the area obtained by land reclamation, there are 10,039 targets, and there are 376 targets with displacement velocity greater than −10 mm/year. More than half of the targets with displacement velocity higher than −10 mm/year are located in the area obtained by land reclamation. There are 44,464 targets in the PSI inversion results, of which 374 targets points with a displacement velocity over −10 mm/year, accounting for 0.8%. There are 1519 targets in the area obtained by land reclamation, and 185 targets with displacement velocity above −10 mm/year (Figure 11c,d). Similarly, more than half of the targets with an annual deformation rate greater than −10 mm/year are located in the land reclamation area. In the sub-region, the target with annual deformation rate greater than −10 mm/year by SBAS and PSI inversion result is mainly located in the area obtained by land reclamation. It is worth noting that PSI results will are sparser due to the stricter screening conditions of the PS points. The area where significant subsidence signals appear in the land reclamation area, combined with satellite optical image information, shows that areas with large surface deformations are mostly located in the area where the underground construction project in progress or recently completed areas. The following three regions that are representative of the region are selected for analysis.

**Figure 12:**
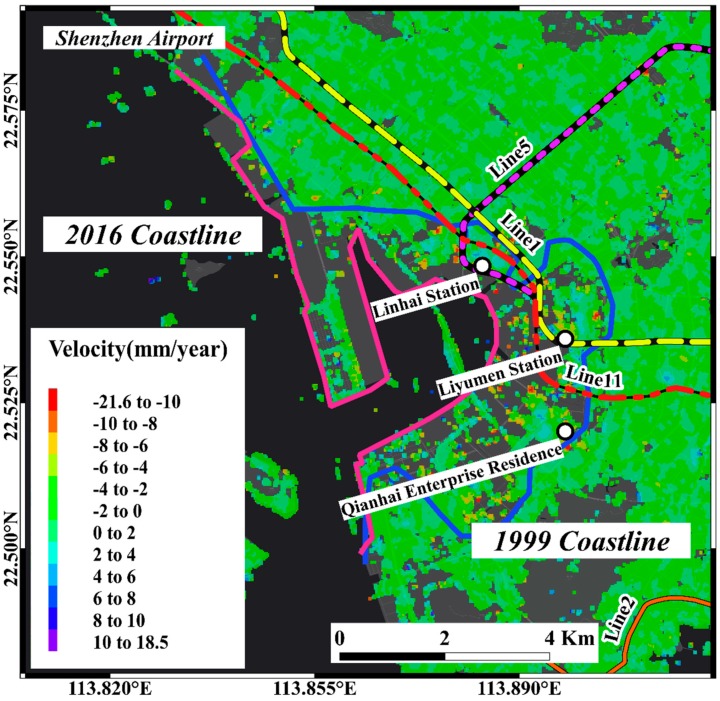
SBAS result of Qianhai bay. The blue line represents the coastline of 1999, and the pink line represents the coastline of 2016.

Seen in Figure 12, the Qianhai Bay of Shenzhen acquired more land for urban construction by various phases of land reclamation projects from 1999 to 2016. The area where the subsidence signal appears in the SBAS results distributed in the land reclamation area that was generated after 1999. It is worth noting that the results of InSAR in these areas are missing because of the lack of effective PS targets in areas where land reclamation was completed in recent years. Moreover, because the conditions for PS searching are stricter than the calibration of ordinary coherent target points, the result point density of PSI inversion in the study area was lower than the SBAS results (Table 2). The three metro lines located in the land reclamation area are Line 1, Line 5, and Line 11. We found that there are a series of areas that appeared as subsidence signals in the area along the subway.

**Figure 13:**
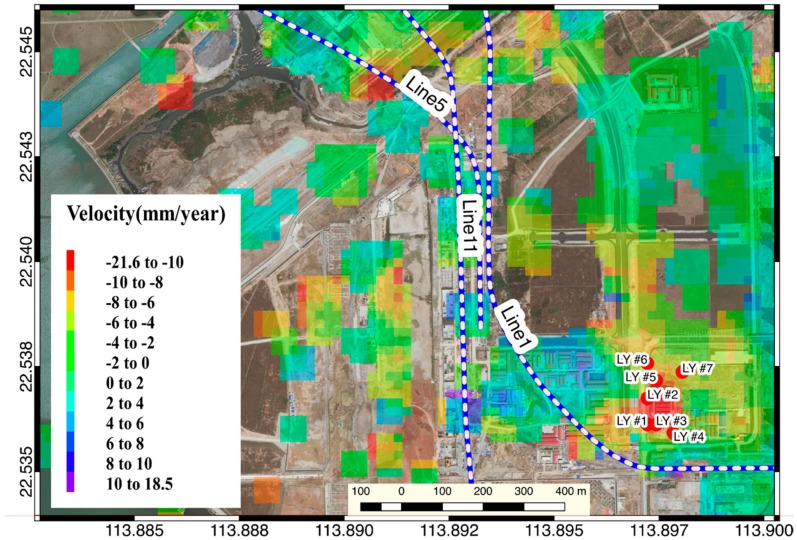
Time series InSAR inversion result of Liyumen station.

**Figure 14:**
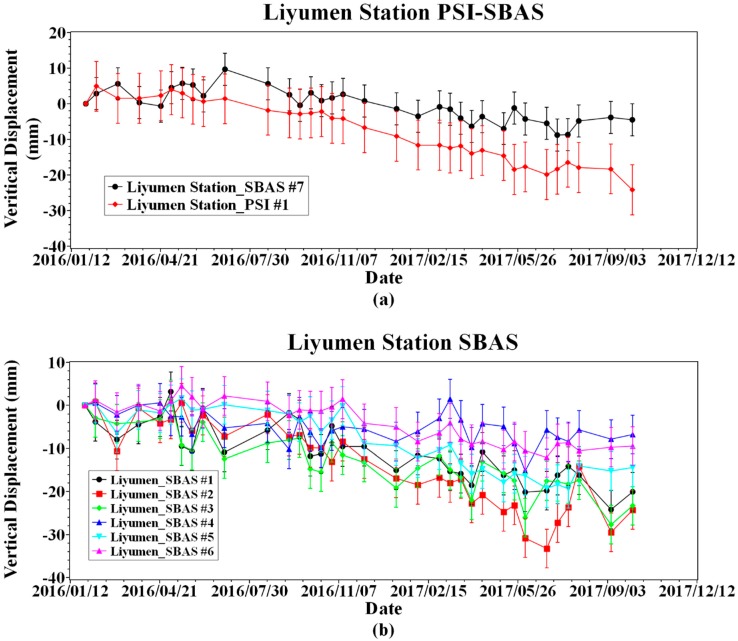
Time-series of sampling point in the Liyumen Station region.

Among them, there is a significant settlement signal on the north side of the Liyumen station of Shenzhen Metro Line 1 (Figure 13). Extracting the time series analysis of pixel deformation in time series InSAR inversion results, we can find that, after November 2016, a trend of continuous subsidence occurred when the cumulative settlement reached 300 mm. On the sample point #5, #6, which is relatively far from the subway line, exhibits a relatively gradual change trend (Figure 14). At the same time, the PSI inversion results show that the PSI #1 sampling points with SBAS #7 sampling points coincide with a relatively uniform subsidence level and subsidence trend. 

**Figure 15:**
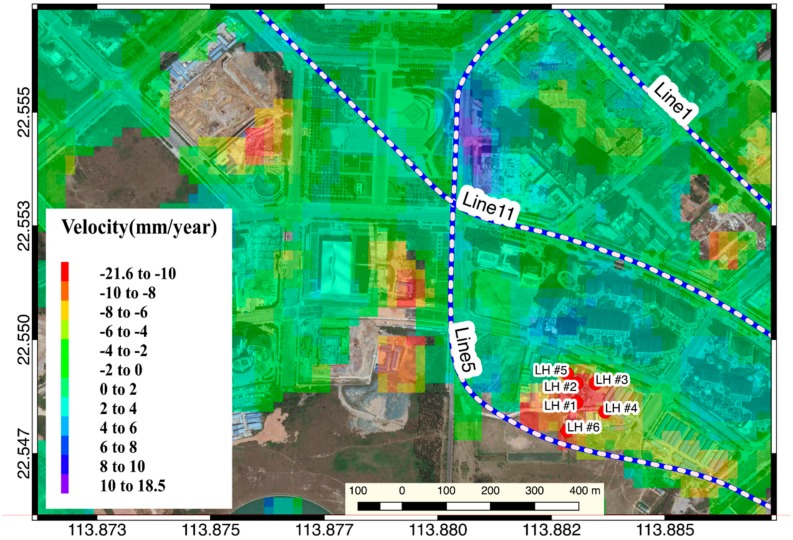
Time series InSAR inversion result of Linhai station.

**Figure 16:**
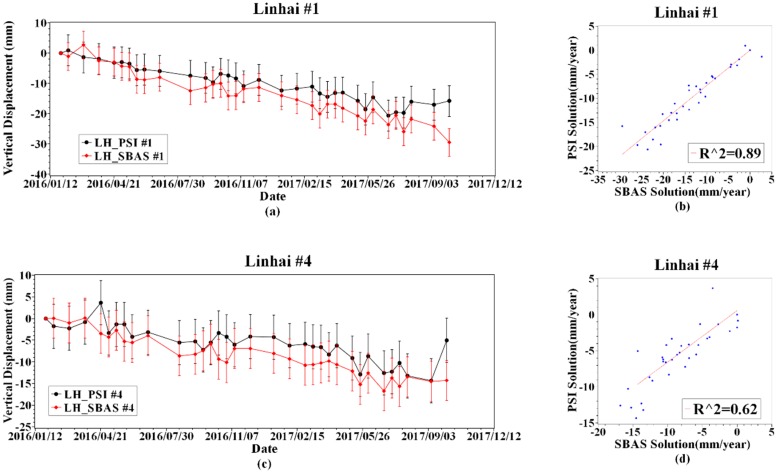

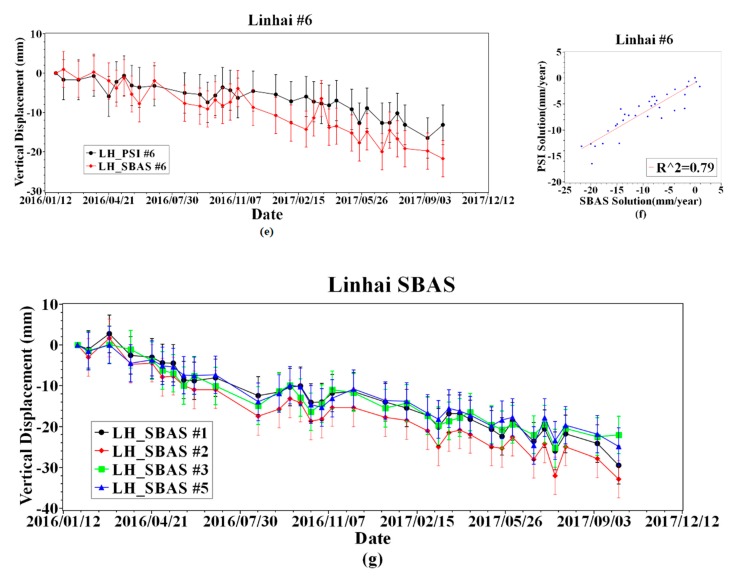
Time-series of sampling point in the Linhai Station region result.

The upper cover of the Linhai station of Shenzhen Metro Line 5, which is also located in Qianhai District, also showed a subsidence zone (Figure 15). The north side of the upper cover of the station in the cross-validation results of the time series InSAR inversion result show a consistent settlement trend throughout the monitoring period. Among them, #6 which is closest to the subway line shows an accelerated subsidence trend after November 2016. Since the PSI results are sparse, there are only sampling points #1, #4, #6 that have both PSI and SBAS inversion results (Figure 16). At these three sampling points, the R^2^ between SBAS results and PSI results can reach 0.6 or higher (Figure 16b,d,f).

**Figure 17:**
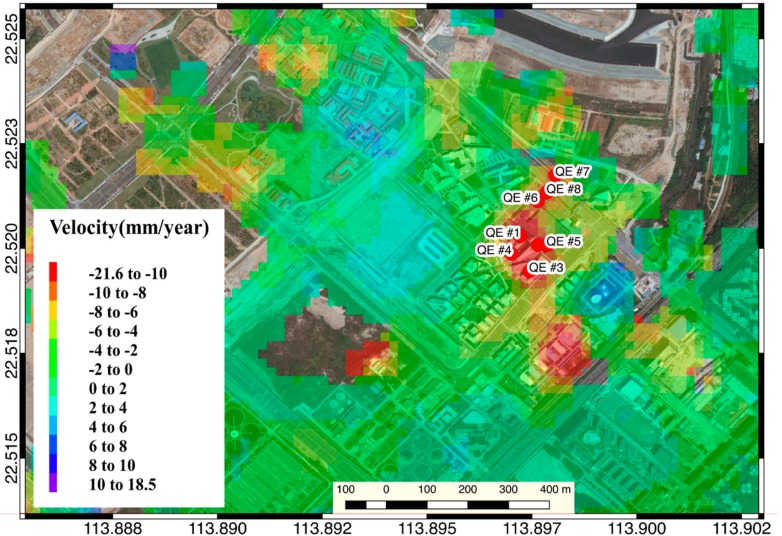
Time series InSAR inversion result of Shenzhen Qianhai Enterprise Residence.

**Figure 18:**
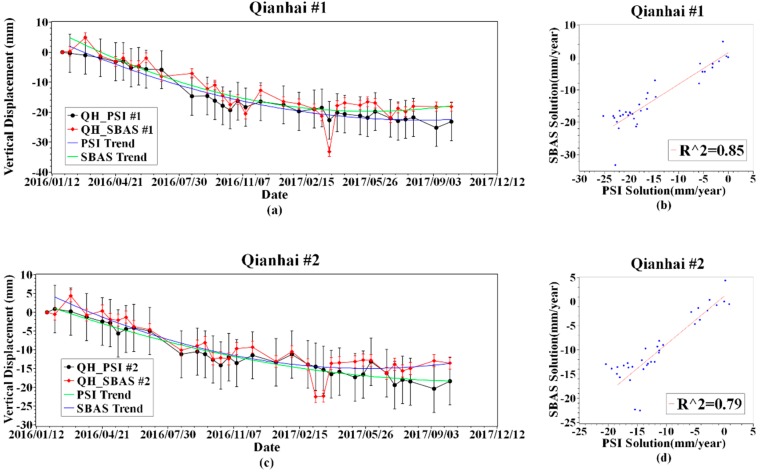

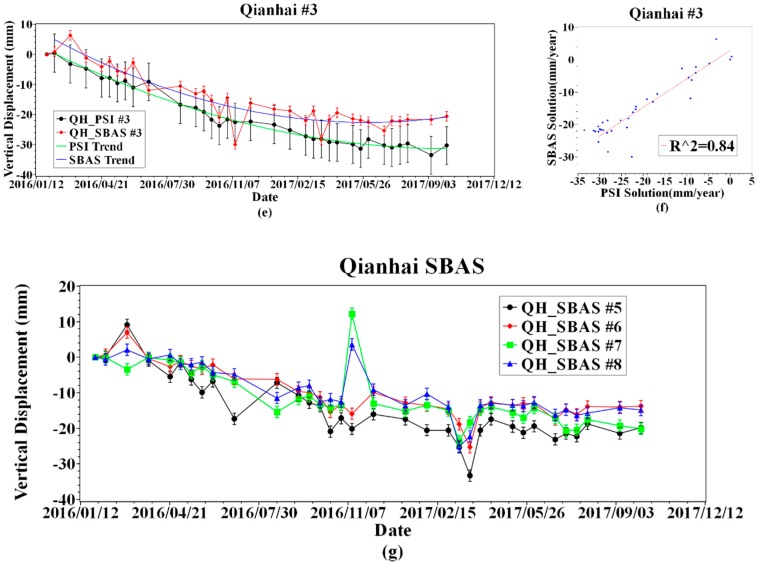
Time-series of sampling point in the Shenzhen Qianhai Enterprise Residence.

The Qianhai Enterprise Residence, completed in 2014, showed subsidence during the entire monitoring period, and the accumulated settlement reached 40 mm (Figure 17). The Qianhai Enterprise Residence is located in the weak formation area. In the results of two different InSAR inversion methods, the main subsidence area occurred at the south side of the building. The coefficient of determination, R^2^ between the PSI result and the SBAS result on the sampling point #1, #2, #3 (Figure 18b,d,f) can reach over 0.79, which shows a high consistency between the PSI and the SBAS results. In the whole monitoring period, the land deformation rate of the settlement trend always tended to be moderate and, considering the completion time, indicates that the area is possible in a self-consolidation situation during the monitoring time. However, due to the active activity of infrastructure activities in the Qianhai area, the landform changes faster which means that the radar reflection signals provided by these type of ground targets cannot maintain high coherence between the master image and the slave image. When using PSI for inversion, fewer PS points were searched. It makes PSI not as good as the SBAS method in terms of spatial resolution in this application scenario. 

## 6. Conclusions

In this research, 34 frames of Sentinel-1A SAR images were used for SBAS and PSI processing, and GNSS observation data were used to perform surface deformation observation of the study area. From the results of data processing, three different methods have the ability to monitor the surface displacement, no matter the station or regional. The results show that the surface deformation obtained by the inversion of SBAS and PSI is highly consistent in magnitude and trend. Both PSI and SBAS show that the subsidence area in the study area was mainly concentrated in Qianhai District, which has recently completed land reclamation activities. In the reclamation area, especially along the underground traffic facilities [33] are the area that the subsidence phenomenon mainly concentrated. However, the TS-InSAR technology, which we mainly focus on, can locate and continuously monitor the areas with subsidence phenomena without prior information through periodic SAR image data. The application of TS-InSAR, especially SBAS technology, for surface deformation inversion has a higher spatial resolution than GNSS technology, hence it has advantages in the subsidence area detection. Since InSAR technology uses time and spatial filtering to remove the APS of each interferogram [1], the results of InSAR inversion are more stable than GNSS calculations. Although the GNSS method can obtain a three-dimensional variation of the station, the GNSS solution results are less accurate in the vertical direction. In the results, the implementation effect in monitoring the vertical displacement is not as good as the InSAR. However, GNSS technology has significant advantages in horizontal displacement monitoring, making it possible to apply GNSS technology to the region where the deformation signal appears in the InSAR result to obtain the horizontal change information of the deformation region. In the InSAR inversion results, land subsidence phenomena occur in areas with frequent human activities, although the current InSAR inversion model in this research does not distinguish well between the foundation trench excavation and the urban surface subsidence signal. From the result, we found that the land reclamation area of Qianhai District began to carry out infrastructure construction within ten years of completion of land reclamation work which with a higher risk. However, due to the extensive development of underground projects, such as subways, and the extensive geological distribution of land reclamation projects and underground caves, it has also increased the risk of the occurrence of surface deformation.

In terms of data processing, we expect to use GNSS at MIT/GLOBal Kalman (GAMIT/GLOBK) software for a higher accuracy baseline solution. In the process of collecting GNSS observation data, it is also possible to add non-synchronous observation data to constitute the asynchronous loop to enhance the reliability of the GNSS static network. With the use of two orbital SAR images in the same region for InSAR data processing, it is possible to acquire three-dimensional variations of coherent targets using InSAR technology.

## Figures and Tables

**Table 1:** Acquisition date and the relative orbital position of each image relative to the master image (2017/4/17) of the Sentinel-1A SAR image we used in this paper.

No.	Acquisition Date	Relative Position (m)	No.	Acquisition Date	Relative Position (m)
1	2016/1/29	164.056	18	2016/12/6	35.492
2	2016/2/10	150.344	19	2017/1/11	123.824
3	2016/3/5	46.199	20	2017/2/4	86.124
4	2016/3/29	27.642	21	2017/2/28	135.102
5	2016/4/22	91.172	22	2017/3/12	89.354
6	2016/5/4	101.625	23	2017/3/24	114.535
7	2016/5/16	59.264	24	2017/4/5	58.529
8	2016/5/28	61.442	25	2017/4/17	0.000
9	2016/6/9	77.456	26	2017/5/11	37.645
10	2016/7/3	53.314	27	2017/5/23	100.012
11	2016/8/20	67.135	28	2017/6/4	38.256
12	2016/9/13	71.373	29	2017/6/28	71.516
13	2016/9/25	15.231	30	2017/7/10	125.536
14	2016/10/7	47.799	31	2017/7/22	118.556
15	2016/10/19	123.272	32	2017/8/3	56.789
16	2016/10/31	106.400	33	2017/9/8	121.120
17	2016/11/12	106.080	34	2017/10/2	64.370

**Table 2:** Inversion results between Permanent Scatterer InSAR (PSI) and Small BAseline Subset InSAR (SBAS).

	Land Reclamation Region (PSI)	All Region (PSI)	Land Reclamation Region (SBAS)	All Region (SBAS)
Target Points	1519	44,464	10,039	255,433
Velocity >−5 mm/y Points	610	6016	1827	9702
Velocity >−10 mm/y Points	185	374	376	702
